# Killer-cell immunoglobulin-like receptors and malaria caused by *Plasmodium falciparum* in The Gambia

**DOI:** 10.1111/j.1399-0039.2011.01818.x

**Published:** 2012-02

**Authors:** L-M Yindom, R Forbes, P Aka, O Janha, D Jeffries, M Jallow, D J Conway, M Walther

**Affiliations:** Medical Research Council LaboratoriesFajara, Banjul, The Gambia

**Keywords:** Gambia, human leukocyte antigen, killer-cell immunoglobulin-like receptor, Malaria, Natural killer cells, *Plasmodium falciparum*

## Abstract

The relevance of innate immune responses to *Plasmodium falciparum* infection, in particular the central role of natural killer (NK) cell-derived interferon gamma (IFN-γ), is becoming increasingly recognised. Recently, it has been shown that IFN-γ production in response to *P. falciparum* antigens is in part regulated by killer-cell immunoglobulin-like receptor (*KIR*) genes, and a study from malaria-exposed Melanesians suggested an association between KIR genotypes and susceptibility to infection. This prompted us to determine and compare the frequencies of 15 *KIR* genes in Gambian children presenting with either severe malaria (*n* = 133) or uncomplicated malaria (*n* = 188) and in cord-blood population control samples (*n* = 314) collected from the same area. While no significant differences were observed between severe and uncomplicated cases, proportions of individuals with *KIR2DS2+C1 and KIR2DL2+C1* were significantly higher among malaria cases overall than in population control samples. In an exploratory analysis, activating *KIR* genes *KIR2DS2*, *KIR3DS1* and *KIR2DS5* were slightly higher in children in disease subgroups associated with the highest mortality. In addition, our data suggest that homozygosity for KIR genotype A might be associated with different malaria outcomes including protection from infection and higher blood parasitaemia levels in those that do get infected. These findings are consistent with a probable role of *KIR* genes in determining susceptibility to malaria, and further studies are warranted in different populations.

## Introduction

Infection with the malaria parasite *Plasmodium falciparum* can result in asymptomatic parasite carriage, an uncomplicated febrile disease or a potentially life-threatening illness. Apart from clinical immunity that gradually develops with repeated exposure (1), human genetic variation influences clinical outcome in response to parasite encounter (2). Epidemiological data from Kenya have indicated that about 25% of the risk of being infected with malaria parasites can be attributed to human genetic variation (3).

Natural killer (NK) cells are a key component of innate immunity. They kill their targets (diseased cells) by means of cytotoxic activity (4) and production of inflammatory cytokines (5). Traditionally, activation of NK cells is explained by the ‘missing self’ hypothesis (6), where the lack of major histocompatibility complex (MHC) class I molecules on infected or malignant cells is sensed by NK cell surface receptors, or activating NK cell receptors interact with stress-induced molecules on the surface of altered cells (4). While most pathogens can activate NK cells, down-regulation of MHC class I molecules is not a common feature of many infectious diseases, and it is now increasingly recognised that most pathogens predominantly activate NK cells via an indirect pathway, with activating signals (both soluble and contact-dependent) being provided by accessory cells, such as dendritic cells, macrophages and/or monocytes (7).

In protozoan infections, rapid production of interferon gamma (IFN-γ) rather than cytotoxicity is the major contribution of NK cells to host defence (8). For malaria, in particular, there is clear evidence that NK-cell-derived IFN-γ, and not cytotoxicity, contributes to protection (9). Human NK cells in peripheral blood mononuclear cell (PBMC) cultures produce IFN-γ within 6 h of parasite encounter (10), which is dependent on the presence of accessory cells. Within an individual the magnitude of the NK cell IFN-γ response is associated with the strength of the signal provided by the accessory cells (11). However, when compared between different individuals, the degree and magnitude of NK-cell-specific IFN-γ production in response to *P. falciparum* displays considerable heterogeneity (10, 12) and was shown to be significantly associated with killer-cell immunoglobulin-like receptor (KIR) genotypes in two studies (13, 14). Thus, in addition to the strength of the signal received from accessory cells, KIR genotypes seem to regulate the degree of IFN-γ produced by KIR-positive NK cell populations.

NK cells can be subdivided further into two subsets, CD56^bright^ and CD56^dim^ expressing cells (15). While CD56^bright^ cells produce more IFN-γ than CD56^dim^ cells, CD56^dim^ cells represent about 80% of the NK cell population (16), thus the vast majority of IFN-γ producing NK cells are the CD56^dim^ population (14). Interestingly, CD56^bright^ cells are KIR negative, whereas the majority of CD56^dim^ cells express KIRs that are highly polymorphic (16, 17). This makes it plausible that KIR–MHC class I interactions might regulate the magnitude of IFN-γ produced by KIR+CD56^dim^ cells.

KIR molecules are glycoproteins encoded by a diverse and compact set of genes on chromosome 19. They are expressed on specialised lymphoid cells mainly NK cells and a subpopulation of γδ T cells and some memory αβ T cells (18). The family comprises 15 functional genes (*KIR2DL1, KIR2DL2, KIR2DL3, KIR2DL4, KIR2DL5A, KIR2DL5B, KIR2DS1, KIR2 DS2, KIR2DS3, KIR2DS4, KIR2DS5, KIR3DL1, KIR3DL2, KIR3DL3* and *KIR3DS1*) and 2 pseudogenes (*KIR2DP1* and *KIR3DP1*). The expression and function of each of these genes influence the expression and function of other members of the gene family (19). KIR molecules are structurally similar with two or three extracellular domains, a transmembrane region and a cytoplasmic tail but can be divided on functional grounds into activating and inhibitory receptors based on the length and composition of their cytoplasmic tails (20). NK cell-mediated activity is dependent on a fine balance between the strengths of the inhibitory and activating signals induced by KIR molecules on the NK cell surface.

Although located on different chromosomes and therefore segregating separately, the coevolving human leukocyte antigen (HLA) and KIR systems (21, 22) encode for molecules with crucial roles in immune modulation of infectious diseases including malaria (23–25). Certain HLA class I molecules are ligands for *KIR*s and their interactions regulate NK cell activity in modulating disease outcomes (23, 26–33). However, the interaction between *HLA* and *KIR* genes in malaria has not been fully established even though certain HLA class I and II alleles (34, 35), and KIR genotypes have independently been associated with malaria clinical outcomes (36).

Whilst there is accumulating evidence favouring an important role of NK cells in *P. falciparum* infection, only a few studies have investigated the role of KIRs in malaria. A study that compared KIR genotypes in Melanesians with and without malaria parasitaemia found evidence for an increased number of activating *KIR* genes in parasitaemic individuals (36). This suggests that KIR diversity may shape the innate response to malaria parasites and probably influences disease outcome. In this study, we compared the proportion of individuals positive for different combinations of activating and inhibitory *KIR* genes among Gambian children with severe or uncomplicated forms of malaria, and cord-blood population control samples to assess whether individual *KIR* genes or genotypes are associated with the occurrence of disease or parasitaemia levels. Given that epidemiological findings have in the past implicated individual HLA class I alleles (e.g. HLA-B*53) (37) with differential susceptibility to malaria infection, we also investigated whether certain KIR–HLA compound genotypes are associated with malaria outcomes.

## Materials and methods

### Study populations

DNA samples were obtained from buccal swabs collected from Gambian children with uncomplicated malaria (>5000 parasites/µl, a temperature of >37.5 °C; UM) and severe malaria [using modified World Health Organization (WHO) criteria (38); SM], who were enrolled between 2006 and 2009 into a study of the pathogenesis of severe malaria described in more detail elsewhere (39, 40). For each patient, a thin and a thick smear were prepared and Giemsa stained. The diagnosis was made by slide microscopy of the thick film at the health centre. The thin smears were subsequently read in the research laboratory, and parasitaemia counts per microlitre were adjusted for the actual number of red blood cells (RBCs) obtained from the full blood count, collected at the same time. Severe disease was further subdivided into severe anaemia (SA), defined as Hb < 6 g/dl; severe respiratory distress (SRD) defined as serum lactate >7 mmol/l; cerebral malaria (CM) defined as a Blantyre coma score ≤2 in the absence of hypoglycaemia or hypovolaemia, with the coma lasting for at least 2 h; and severe prostration (SP) defined as inability to sit unsupported (children > 6 months) or inability to suck (children ≤ 6 month). For some analyses, different entities of severe diseases were stratified according to increasing disease severity, ranging from UM< SP< SA< CM< SRD< CM+SRD, consistent with a large study from Kenya showing that mortality increases in this order (41). DNA extracted from cord-blood samples of Gambian neonates collected from health-care facilities of the same area for a different study (42) served as population control samples. Both studies were approved by the Joint Gambian Government/MRC Ethics Committee (GGMEC), and written informed consent was obtained from a parent or legal guardian prior to sample collection. For the work described herein, a separate approval was obtained from the GGMEC.

In total, DNA samples from 635 individuals were analysed in this study (314 cord-blood samples, 188 uncomplicated and 133 severe malaria cases). Further demographic details on study participants are given in Table 1.

**Table 1:** Characteristics of the study participants

	*n*	Mortality (%)	Age (GM, years)	95% CI	Female (%)
Severe	133	6.0	4.4	4.01–4.72	42.5
CM+SRD	14	28.6	4.3	3.42–5.38	50.0
SRD	16	18.8	4.7	3.66–5.99	33.3
CM	23	0.0	4.3	3.59–5.06	38.9
SA	11	0.0	2.8	2.02–3.95	45.5
SP	69	1.5	4.6	4.11–5.22	43.8
Uncomplicated	188	0.0	6.3	5.80–6.90	43.1
Cord blood (population control)	314	—	—	—	50.2

CI, confidence interval; CM, cerebral malaria; GM, geometric mean; *n*, number of individuals; SA, severe anaemia; SP, severe prostration; SRD, severe respiratory distress.

### KIR typing

Genomic DNA samples were typed by the polymerase chain reaction-sequence-specific-priming (PCR-SSP) technique as described elsewhere (43) to detect the presence of 14 *KIR* genes: *2DL1, 2DL2, 2DL3, 2DL4, 2DL5, 2DS1, 2DS2, 2DS3, 2DS4, 2DS5, 3DL1, 3DL2, 3DL3, 3DS1,* and 1 pseudogene *KIR2DP1*. Briefly, this technique entailed the use of specific primers to amplify two segments of different sizes from the same *KIR* gene if present. The fragments were then stained with ethidium bromide during electrophoresis in 2% agarose gel. Specific bands were visualised on a UV light box, an electronic picture of the gel was taken and scored for the presence or absence of specific bands. Discrepant results (i.e. one primer pair positive while the other is negative) were repeated and the gene considered present if one of the reaction pairs was consistently positive. The use of two pairs of primers to detect the same gene was to limit false negative results as much as we can in this population that has not been typed for *KIR* genes before. The absence of specific bands on both reactions was confirmed by repeating the typing to make sure that the gene was actually absent. Each reaction also contained a pair of internal control primers amplifying a 796-bp fragment from the third intron of *HLA-DRB1* gene to check for PCR efficiency.

### HLA class I typing

Genotyping for HLA class I (HLA-B and -C) alleles was performed using sequence-based techniques on 148 and 382 samples, respectively as described elsewhere (33). Briefly, a pair of locus-specific primers was used to amplify each locus and two other pairs of internal primers were used to sequence exons 2 and 3 in both directions using the BigDye Terminator version 3.1 technologies (Applied Biosystems, Foster City, CA). The software ‘assign’ (Conexio Genomics, Australia) was used to analyse all sequence traces.

### Statistical analysis

The observed frequency for each *KIR* gene was determined by direct counting and verified using stata version 9.2 (Stata Corporation, TX) and pasw Statistics 18 (SPSS, Inc., Chicago, IL). This corresponded to the proportion of individuals that carried the gene of interest in the group under investigation. HLA class I allele and genotype frequencies were computed with the same statistical packages. The centromeric (cen) and telomeric (tel) motifs of each KIR genotype were assigned using a modified technique derived from Cooley et al. (44) and Pyo et al. (45). Their frequencies and those of *HLA* and *KIR* genes, as well as KIR–HLA compound genotypes were determined and compared across groups (population control, uncomplicated and severe malaria) using chi-squared or Fisher's exact tests as appropriate. *P*-values of <0.05 were considered significant. Correction for multiple comparisons was performed using the Bonferroni method. In addition, adjustment for ethnicity was performed for comparisons between the cases and controls. Differences in parasitaemia levels for groups of individuals with varying ratios of inhibitory over activating *KIR* genes were examined using one-way analysis of variance (anova) on log transformed parasitaemia data, with a post-test for linear trend.

The statistical packages stata version 9.2 (Stata Corporation, TX), pasw Statistics 18 (SPSS, Inc., Chicago, IL) and prism version 5.04 (GraphPad Software Inc., CA) were used to perform all statistical analyses.

## Results

### The frequency of individual *KIR* genes does not differ between ethnicities

All 15 *KIR* genes investigated were present in the study population. Recognising the ethnic diversity of this population, we stratified the observed frequency of each *KIR* gene according to the major ethnic groups. Data for self-reported ethnicity were available for 94% of all samples tested. Table 2 shows that the proportions of individuals carrying any of the *KIR* genes were similar across all ethnic groups. Homogeneity of frequencies among different ethnic groups was formally assessed using the chi-squared distribution or Fisher's exact test and no significant differences were observed. This indicates that ethnic differences are not major determinants of the frequencies here.

**Table 2:** *KIR* frequencies in the study population stratified by ethnicity^a^

*KIR* gene	Mandingo (199) (%)	Wollof (88) (%)	Fula (137) (%)	Jola (89) (%)	Serere (29) (%)	Others (57) (%)	P value
*2DL1*	100.0	100.0	100.0	100.0	100.0	100.0	n.a.
*2DL2*	76.9	69.3	73.7	78.7	58.6	84.2	0.090
*2DL3*	90.0	90.9	88.3	83.2	89.7	77.2	0.097
*2DL4*	100.0	100.0	100.0	100.0	100.0	100.0	n.a.
*2DL5*	60.3	53.4	59.1	56.2	55.2	64.9	0.773
*2DS1*	21.1	23.9	21.2	24.7	24.1	26.3	0.944
*2DS2*	59.8	56.8	58.4	64.0	51.7	70.2	0.492
*2DS3*	43.2	42.1	44.5	49.4	31.0	56.1	0.266
*2DS4*	100.0	100.0	100.0	100.0	100.0	100.0	n.a.
*2DS5*	28.1	25.0	32.8	25.8	34.5	42.1	0.229
*3DL1*	100.0	100.0	100.0	100.0	100.0	100.0	n.a.
*3DL2*	100.0	100.0	100.0	100.0	100.0	100.0	n.a.
*3DL3*	100.0	100.0	100.0	100.0	100.0	100.0	n.a.
*3DS1*	11.1	5.7	7.3	7.9	13.8	10.5	0.578
*2DP1*	99.5	98.9	100.0	98.9	96.6	96.5	0.194 F

n.a., not applicable.

a Information on ethnicity was available for 94% of all samples. Numbers in parenthesis represent the number of individuals per ethnic group. Homogeneity of the frequencies of *KIR* genes among different ethnic groups was assessed using the chi-squared test or Fisher's exact test (*F*) in the case of 2DP1.

### Effect of *KIR* gene in severe and uncomplicated malaria

**Figure 1:**
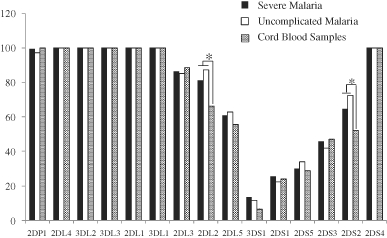
Killer-cell immunoglobulin-like receptor (*KIR*) frequency in uncomplicated and severe malaria cases, and in cord-blood population control samples. Next to the pseudo gene *KIR2DP1*, the frequencies of inhibitory and activating *KIR* genes are shown in decreasing and increasing frequencies, respectively. Differences that remained significant after Bonferroni correction for multiple comparisons are indicated with *.

We next investigated whether proportions of individuals with each of these *KIR* genes differed between severe and uncomplicated malaria cases or between malaria cases and population controls. Figure 1 shows the carrier frequency for each *KIR* gene in the two disease groups and population control samples, and Table S1 (*Supporting Information*) shows the proportion of carriers by disease entities. In addition to the framework genes *KIR2DL4, 3DL2* and *3DL3*, three other genes *KIR2DL1, 2DS4* and *3DL1* were present in all individuals. Overall, proportions of individuals with each of the inhibitory *KIR* genes exceeded 75% with the exception of *KIR2DL5* (58.9%) while those carrying any of the activating *KIR* genes (except *KIR2DS2*) ranged from 9.5% to 45.4%.

Assessment of the homogeneity of proportions positive for each of the *KIR* genes across groups identified significant differences for *KIR2DL2, KIR2DS2, KIR3DS1* and the pseudogene *KIR2DP1*, with their respective *P*-values being 2 × 10^−7^, 2.7 × 10^−5^, 2.7 × 10^−2^ (chi-squared test), and 8 × 10^−3^ (Fisher's exact test), of which only *KIR2DL2* and *KIR2DS2* remained significant after Bonferroni correction for multiple comparison (corrected *P*- values 3 × 10^−6^ and 4.05 × 10^−4^, respectively). Further analysis showed that none of the frequencies differed between uncomplicated and severe malaria cases. However, when all cases (severe and uncomplicated malaria combined) were compared with cord-blood population samples, *KIR2DL2, KIR2DS2* and *KIR3DS1* were significantly more common in cases than in cord-blood samples (chi-squared test, *P* = 1 × 10^−6^, 1.2 × 10^−5^, 8.6 × 10^−3^, respectively), while the pseudogene *KIR2DP1* was slightly less common in cases (Fisher's exact test, *P* = 0.03). After correction for multiple comparisons using the Bonferroni method only *KIR2DL2* and *KIR2DS2* genes remained significantly more frequent in malaria cases than in population controls (corrected *P* < 0.0001 for both comparisons), while the result for *KIR3DS1* and the pseudogene *KIR2DP1* became non-significant (corrected *P* = 0.129 and *P* = 0.45, respectively).

### Relationship between centromeric and telomeric KIR motifs and genotypes with malaria

The typing technique used in this study (PCR-SSP) allowed us to detect the presence of inhibitory and activating *KIR* genes but not their different alleles. On the basis of SSP typing, two KIR genotypes (A and B) have been defined depending on the number and type of haplotypes making the genotype (46). Each KIR genotype is made up of two parts (one centromeric and one telomeric motif) separated by a recombination hot spot area upstream of *KIR2DL4* gene. KIR genotype A has a uniform gene content with its centromeric part containing specific genes such as *KIR2DL3, 2DL1* and *2DP1,* in addition to the frame work genes *3DL3* and *3DP1.* The telomeric part of KIR genotype A is marked by the presence of *KIR3DL1* and *2DS4* and the ubiquitous *KIR2DL4* and *KIR3DL2* (frame work genes). Individuals homozygous for KIR genotype A have two copies of A haplotypes [i.e. two copies of centromeric A motifs (c-A/A) and two telomeric A motifs (t-A/A)].

In contrast to the conserved nature of genotype A in terms of gene content, KIR genotype B is highly polymorphic in gene content. Genotype B is a combination of activating and inhibitory *KIR* genes including those found in genotype A. The number of specific genes found at the centromeric end of B genotypes is variable including *KIR2DS2, 2DL2, 2DL5, 2DS3* and *2DS5*, in addition to the framework genes *3DL3* and *3DP1.* However, telomeric B motifs are specifically identified with the presence of *KIR3DS1* and *2DS1* genes in addition to *2DL4* and *3DL2* present in most motifs.

We adopted the recent techniques and terminologies used by Cooley et al. (44) and Pyo et al. (45) to assign centromeric (cen) and telomeric (tel) motifs and genotypes to each of our samples. The most frequent KIR genotypes included c-AB2/ t-AA (17.32%) and c-AA/t-AA (16.7%) (not shown). Individuals with c-AB2/t-AA genotype are heterozygotes for A and B2 motifs at the centromeric part while their *KIR* genes at the telomeric end all belong to KIR haplotype A (Figure S1). On the other hand, carriers of c-AA/t-AA genotype are homozygotes for A haplotypes meaning they lack all activating *KIR* genes except *KIR2DS4* (Figure S1). Analysis of KIR centromeric and telomeric genotype data (shown in Table 3, and Figure S1) did not show significant differences between uncomplicated and severe malaria cases. Similarly, exploratory analyses comparing KIR genotype frequencies amongst different disease entities (uncomplicated malaria, SP, SA, CM, SRD and CM+SRD) showed no significant associations between KIR genotypes and malaria severity (Table S1). However, comparing the three groups together showed a significant imbalance in the distribution of individuals homozygous for centromeric A motifs, with a higher proportion of carriers found in the cord-blood population control group than in cases (*P* = 0.001; adjusted for ethnicity and multiple comparisons *P* = 0.007). An opposite effect was observed for heterozygote (c-A/B) carriers (Table 3), with a higher proportion of carriers found amongst malaria cases (*P* = 0.002, adjusted for ethnicity and multiple comparisons *P* = 0.021). No such differences were seen with telomeric genotypes.

**Table 3:** Effect of centromeric and telomeric motifs and KIR genotypes on malaria

Genotype	Uncomplicated malaria *n* (%)	Severe malaria *n* (%)	Combined cases *n* (%)	Pop control *n* (%)	*P*_A_	*P*_B_	*P*_C_	OR (95% CI)
Centromeric								
c-A/A	20 (10.6)	20 (15.0)	40 (12.46)	69 (22.0)	0.004	0.001	0.007	0.5 (0.32–0.79)
c-B/B	31 (16.5)	19 (14.3)	50 (15.58)	56 (17.8)	0.652	0.446	ns	0.92 (0.59–1.44)
c-A/B	137 (72.9)	94 (70.7)	231 (71.96)	189 (60.2)	0.007	0.002	0.021	1.61 (1.14–2.29)
Telomeric								
t-A/A	135 (71.8)	90 (67.7)	225 (70.09)	231 (73.6)	0.448	0.331	ns	1.01 (0.7–1.45)
t-A/B	53 (28.2)	43 (32.3)	96 (29.91)	83 (26.4)	0.448	0.331	ns	0.99 (0.69–1.43)
KIR								
A/A	20 (10.6)	20 (15.0)	40 (12.46)	66 (21.0)	0.009	0.004	0.012	0.53 (0.34–0.84)
B/x	168 (89.4)	113 (85.0)	281 (87.54)	248 (79.0)	0.009	0.004	0.012	1.87 (1.19–2.96)

Bx, non-A centromeric or telomeric part that is different from known B motifs; CI, 95% confidence intervals; *n*, number of individuals positive for the genotype of interest; P, chi-squared *P*-values comparing (A) all groups, (B) combined cases (uncomplicated and severe malaria) *vs* population controls, and (C) as for B above but corrected for multiple comparisons by the Bonferroni approach after adjustment for ethnicity; ns, non-significant; OR, odds ratios for cases *vs* controls of having a particular genotype; Pop control, cord blood population control samples.

The proportion of individuals homozygous for KIR genotype A varied significantly amongst the three groups (Table 3). Whilst no significant difference was observed between severe and uncomplicated cases, the proportion of individuals carrying the A/A genotype in the population control group was almost double that in the infected group (21.0% *vs* 12.5%, respectively, *P* = 0.004; adjusted for ethnicity and multiple comparisons *P* = 0.012), suggesting that having two copies of haplotype A may be protective against malaria infection. An interesting observation was that none of the 14 children with CM+SRD, considered to be the most critically ill patients, was homozygous for KIR genotype A, while a substantial proportion of children with less severe forms of malaria were homozygous for genotype A (11%, 16%, 18% and 26% in UM, SP, SA and CM, respectively). Given the small number of individuals per group, this should be considered an exploratory analysis that encourages further investigations in other populations with larger sample sizes.

### KIR and HLA-C alleles show significant association with malaria infection and severity

We sequenced HLA-C and -B alleles from 382 and 148 samples, respectively, and compared their frequencies across groups as shown in Table 4. We found HLA-Cw*16:01 frequency (a C group 1 allele) to be lowest amongst children with severe malaria (*P* = 0.007, for comparison across three groups, *P* = 0.077 after adjustment for multiple comparisons). This observation, however, should be taken with caution given the small number of individuals used in this comparison. None of the HLA-B alleles was found to have any impact on malaria in this population. The frequencies of HLA-B or -C dimorphic groups and subgroups (Bw4, Bw6, Bw4-80I, Bw4-80T, C1, C2, C1/C2) were similar between groups (Table 4).

**Table 4:** HLA alleles and KIR–HLA compound genotypes on malaria^a^

	Malaria cases			
			
Allele*	Uncomplicated (%)	Severe (%)	Pop controls	*P*
HLA-C				
Cw^*^02	16 (16.3)	13 (22.4)	52 (23.0)	0.389
Cw^*^03	22 (22.5)	22 (37.9)	56 (24.8)	0.079
Cw^*^04	35 (35.7)	14 (24.1)	68 (30.1)	0.305
Cw^*^05:01	8 (8.2)	3 (5.2)	8 (3.5)	0.205 F
Cw^*^06:02	11 (11.2)	7 (12.1)	25 (11.1)	0.977
Cw^*^07	21 (21.4)	16 (27.6)	60 (26.6)	0.571
Cw^*^08	4 (4.1)	5 (8.6)	12 (5.3)	0.486 F
Cw^*^15	7 (7.1)	3 (5.2)	7 (3.1)	0.224 F
Cw^*^16:01	30 (30.6)	5 (8.6)	55 (24.3)	0.007
Cw^*^17	7 (7.1)	8 (13.8)	26 (11.5)	0.363
Cw^*^18	6 (6.1)	2 (3.5)	7 (3.1)	0.422 F
HLA-B				
B^*^07	6 (14.0)	3 (11.5)	4 (5.1)	0.217 F
B^*^08:01	3 (7.0)	2 (7.7)	7 (8.9)	1.000 F
B^*^15	10 (23.3)	6 (23.1)	25 (31.7)	0.518
B^*^18:01	1 (2.3)	1 (3.9)	3 (3.8)	1.000 F
B^*^35:01	12 (27.9)	5 (19.2)	16 (20.3)	0.583
B^*^52:01	3 (7.0)	2 (7.7)	6 (7.6)	1.000 F
B^*^53	12 (27.9)	7 (26.9)	30 (38.0)	0.403
B^*^58	7 (16.3)	5 (19.2)	13 (16.5)	0.940
B^*^78	4 (9.3)	3 (11.5)	9 (11.4)	1.000 F
Group				
Bw4	28 (65.1)	16 (61.5)	56 (70.9)	0.623
Bw4-80I	27 (62.8)	16 (61.5)	43 (54.4)	0.621
Bw6	32 (74.4)	18 (69.2)	58 (73.4)	0.888
C1	76 (77.6)	42 (72.4)	173 (76.6)	0.751
C2	70 (71.4)	36 (62.1)	144 (63.7)	0.343
C1C2	48 (49.0)	20 (34.5)	91 (40.3)	0.417
KIR–HLA				
3DS1+Bw4	5 (11.6)	3 (11.5)	3 (3.8)	0.148 F
3DL1+Bw4	28 (65.1)	16 (61.5)	56 (70.9)	0.623
3DL1+Bw4-801	27 (62.8)	16 (61.5)	43 (54.4)	0.621
2DL2+C1	66 (67.4)	38 (65.5)	101 (44.7)	**<0.001**
2DL3+C1	63 (64.3)	38 (65.5)	154 (68.1)	0.777
2DS2+C1	54 (55.1)	33 (56.9)	77 (34.1)	**<0.001**
2DL1+C2	70 (71.4)	36 (62.1)	144 (63.7)	0.343
2DS1+C2	17 (17.4)	11 (19.0)	31 (13.7)	0.512

P, chi-squared or, where indicated, Fisher exact *P*-values comparing the three groups, *P*-values that remained significant after adjustment for ethnicity and correction for multiple comparisons (Bonferroni) are printed in bold; ^*^, two-digits results represent alleles with more than one subtypes; Pop controls, population-based control samples.

a Numbers before the parenthesis indicate the number of individuals positive for the genotype of interest. Rare alleles (present in less than one percent of the studied population) are not included. HLA-C analysis was based on data from 382 samples and HLA-B data was available for 148 individuals.

It has been shown that KIR and HLA interact in an epistatic manner to modulate human immunodeficiency virus (HIV) disease outcome (29). In this study, we grouped individuals based on whether they have corresponding putative ligand(s) for their *KIR* genes. Analysis of KIR–HLA compound genotypes as given in Table 4 showed significant differences in the proportion of individuals carrying either *KIR2DL2* and/or *KIR2DS2* together with their corresponding ligands [HLA-C group 1 (C1)]. The frequencies of these two compound genotypes were significantly higher in cases compared with population control (*P*≤ 2 × 10^−4^; adjusted for ethnicity and multiple testing *P*≤ 0.0008 for both comparisons), suggesting that carriers of *KIR2DL2*+*C1* and/or *KIR2DS2*+*C1* were more at risk of being infected with malaria parasites than those without any of these genotypes. However, these two genes are in strong linkage disequilibrium (LD) even in African populations (47, 48) making it difficult to know which of them is actually mediating the effect. As earlier mentioned, future studies are needed to confirm this preliminary observation.

### The ratio of inhibitory to activating *KIR* genes and the number of B motifs per KIR (B content) are associated with level of parasitaemia

**Figure 2:**
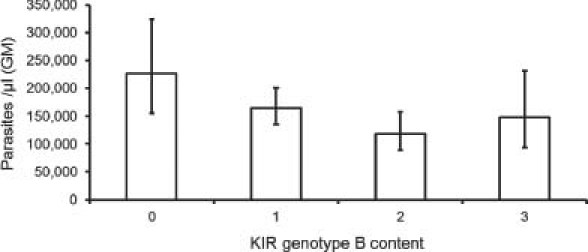
Geometric mean parasitaemia levels according to the B content of killer-cell immunoglobulin-like receptor (KIR) genotypes. Error bars represent the 95% confidence interval (CI). Linear regression on log transformed parasitaemia data indicates that parasitaemia declines with increasing B content (*P* = 0.018, coefficient: −0.22 (95% CI: −0.4 to −0.04).

NK cell-derived IFN-γ has been proposed as an important player of the innate immune response to malaria (10), contributing to initial parasite control (49). Considering that expression of KIR receptors on NK cells has been associated with the magnitude of IFN-γ responses to malaria parasites (14), we explored whether the ratio of inhibitory over activating *KIR* genes (I/A ratio) or the carriage of a particular KIR genotype is associated with parasite load measured on admission. When the ratios were grouped into four bins containing similar numbers of individuals, the level of parasitaemia was significantly different (*P*_ANOVA_ = 0.03) showing a significant increase with increasing I/A ratio (*P* test for linear trend = 0.009, Figure S2a). Further, individuals homozygous for KIR genotype A (A/A) had a 1.6-fold higher geometric mean parasitaemia (Figure S2b) than those with one or more B motif content (B/x) (*P* = 0.04), and parasitaemia levels declined significantly with increasing B content of the KIR genotype (*P* = 0.018, Figure 2).

## Discussion

In this study, we determined the presence or absence of 15 *KIR* genes in two groups of children infected with the malaria parasite *P. falciparum* with distinct disease outcomes (severe malaria *vs* uncomplicated malaria) and in a population control group. HLA class I (B and C) genotyping was performed on a proportion of samples with enough genomic DNA left after KIR typing. Possible KIR–HLA interactions were explored as compound genotypes in individuals expressing KIR molecules together with their putative ligand(s). Consistent with previous studies in other West African populations (33, 48), we found that the frequencies of inhibitory *KIR*s were significantly higher overall compared with those of activating *KIR* genes across disease entities and major ethnic groups represented in the studied population. While the frequency of individual *KIR* genes did not differ between severe and uncomplicated cases, carriers of *KIR2DL2*, *KIR2DS2* genes were more frequent amongst cases (SM and UM combined) compared with the population control group. Amongst the participants with available HLA type individuals carrying either *2DL2* or *2DS2* together with their corresponding ligand (HLA-C group 1) were significantly more frequent in the infected group. Taken together, these observations suggest that these genes could be involved in increased susceptibility to malaria infection caused by *P. falciparum*. Although a few of our study participants have one without the other, *KIR2DL2* and *2DS2* genes are in strong LD in most populations worldwide including those of African origin (33, 48, 50). Because of the strong LD between the two loci, it is difficult to separate the effect of one locus from the other. The inhibitory *KIR* gene (*2DL2)* and its corresponding activating counterpart (*KIR2DS2*) have been associated with other diseases. Absence of these genes has been associated with resistance to infection with herpes simplex virus type-1 (HSV-1) (51). The presence of *KIR2DS2* in the absence of its specific ligands has been associated with increased susceptibility to psoriatic arthritis (30). On the other hand, when both genes are present together with their appropriate HLA-C ligands, they were associated with reduced risk of chronic myeloid leukaemia (31).

Although the proportion of subjects with individual *KIR* genes did not differ significantly between disease subgroups, it is worth noting that prevalence of three activating *KIR* genes, *KIR2DS2, KR2DS5* and *KIR3DS1* were highest in the most critically ill children presenting with CM+SRD (Table S1). While the relatively low number of children in each group precludes firm conclusions, this observation is in line with the finding that the predominantly inhibitory KIR genotype A is virtually absent in the two groups of severe cases (SRD and CM+SRD) that bear the highest mortality. Our data also suggest that homozygosity for KIR genotype A (A/A) might be protective against malaria infection, and that this effect could mainly be associated with A/A homozygosity at the centromeric segment of the KIR locus. Clearly, larger studies are required to establish whether susceptibility to malaria infection and/or mortality from severe disease is associated with a higher frequency of a particular combination of activating *KIR* genes or a relative absence of inactivating *KIR* genes. If confirmed, this observation might be explained by the concept of NK cell licensing (52), arming (53) or education (54), whereby both tolerance to healthy cells as well as the strength of the response of a mature NK cell towards an infected cell are determined by the interactions between MHC class I molecules and at least one expressed inhibitory KIR. Accordingly, NK cells that show the strongest inhibition from attacking healthy cells will most probably mount the strongest responses towards infected cells (55). However, considering that individuals homozygous for haplotype A (thus carrying the ‘inhibitory’ KIR genotype A/A) produced significantly less IFN-γ in response to stimulation with *P. falciparum* antigens (14) it is plausible too that the predominance of activating *KIR*s (or the relative absence of inhibitory *KIR*s) contributes to exacerbated inflammatory responses that have been implicated repeatedly in the pathogenesis of severe malaria (13, 56). But, as our data suggest, the protective effect of a predominantly inhibitory KIR (A/A genotype) may come at the price of impaired NK-cell-mediated parasite clearance during the early phase of infection.

Present in 6.4% and 12.5% of our population control samples and malaria cases respectively, *KIR3DS1* was the least frequent *KIR* gene in the Gambian population, in line with previous data from other African populations (48, 50, 55). Comparison of *KIR* gene evolution amongst different populations worldwide has suggested that *KIR3DS1/L1* has been under positive selection in modern Sub-Saharan African populations, with *KIR3DS1* being rare and *KIR3DL1* allotypes predominating (55). This would imply a biological benefit derived from a low frequency of *KIR3DS1*, such as reduced susceptibility to malaria or protection from severe forms of malaria, for which our data provide some support. In a study that compared KIR genotypes in Melanesian individuals with and without malaria parasitaemia living in a malaria hyper-endemic area *KIR3DS1* positivity was equally distributed in both groups, but *KIR3DS1/L1* heterozygosity was significantly associated with being parasite positive.

Taken together, our data provide some support for the hypothesis that *KIR* genes influence susceptibility to malaria. The involvement of genes located at the centromeric part of the KIR locus in modulating the outcome of malaria and in particular the role of *KIR2DL2* and *KIR2DS2* in the susceptibility to *P. falciparum* malaria warrant further investigations, and we recommend that larger studies should be performed across different populations to test this hypothesis with additional power. Such studies are needed to complement genome-wide approaches (42) that use single nucleotide polymorphism analysis which is not designed to capture the extensive *KIR* haplotypic diversity.

## Supporting information

The following supporting information is available for this article:

**Figure S1.** The KIR centromeric (A) and telomeric (B) genotypes and their motif content. Filled box indicates that gene is present and open box indicates that the gene is absent. c-A: centromeric A motif, c-B: one of the known centromeric B motifs, c-Bx: one of the new B motifs. UM: uncomplicated malaria, n, number of individuals carrying the genotype of interest; SM, severe malaria. Rare genotypes present in less than 1% of the studied population are not included.

**Figure S2.** (a) Ratio of inhibitory/activating *KIR*genes and (b) KIR genotypes associate with level of parasitaemia inthe blood. Bars represent the geometric mean with 95% CI, n, numberof individuals with blood parasitaemia data, *P*-value in (a)refers to the test for linear trend and *P*-value in (b)refers to *t*-test, both performed on log transformeddata.

**Table S1.** *KIR* distribution in the malaria infected group by disease entities.
