# Supplementary material for: Killer-cell immunoglobulin-like receptors and malaria caused by Plasmodium falciparum in The Gambia
Source: Tissue Antigens. 2012 Feb;79(2):104–13. doi: 10.1111/j.1399-0039.2011.01818.x (PMC3320664; doi:10.1111/j.1399-0039.2011.01818.x)
Supplement: Supplementary file 1 [file tan0079-0104-SD1.pdf]

Killer-cell Immunoglobulin-like Receptors (*KIR*) and malaria caused by *Plasmodium falciparum* in The Gambia

Louis-Marie Yindom[1*], Robert Forbes[1], Peter Aka[1], Omar Janha[1], David Jeffries[1], Muminatou Jallow[1], David J. Conway[1], Michael Walther[1]

Medical Research Council Laboratories, Fajara, Banjul, The Gambia[1]
E-mail: louis-marie.yindom@ndm.ox.ac.uk

Journal: Tissue Antigens

Supplementary Table 1 *KIR* distribution in the malaria infected group by disease entities

KIR gene	UM (188) (%)	SP (69) (%)	SA (11) (%)	CM (23) (%)	CM+SRD (14) (%)	SRD (16) (%)	P value
2DL1	100.0	100.0	100.0	100.0	100.0	100.0	n.a.
2DL2	87.2	79.7	81.8	69.6	92.9	93.8	0.159 F
2DL3	85.1	84.1	100.0	95.7	71.4	87.5	0.313 F
2DL4	100.0	100.0	100.0	100.0	100.0	100.0	n.a.
2DL5	62.8	62.3	63.6	43.5	71.4	68.8	0.514
2DS1	22.3	29.0	9.1	17.4	28.6	31.3	0.585
2DS2	72.3	66.7	63.6	52.2	85.7	56.3	0.186 F
2DS3	42.0	50.7	36.4	30.4	50.0	50.0	0.554
2DS4	100.0	100.0	100.0	100.0	100.0	100.0	n.a.
2DS5	34.0	29.0	36.4	26.1	50.0	18.8	0.488
3DL1	100.0	100.0	100.0	100.0	100.0	100.0	n.a
3DL2	100.0	100.0	100.0	100.0	100.0	100.0	n.a.
3DL3	100.0	100.0	100.0	100.0	100.0	100.0	n.a.
3DS1	11.7	8.7	18.2	8.7	28.6	25.0	0.202
2DP1	97.3	98.6	100.0	100.0	100.0	100.0	1.0 F

The proportion of children carrying any of the 15 individual *KIR* genes are shown for uncomplicated malaria (UM), severe prostration (SP), severe anaemia (SA), cerebral malaria (CM), severe respiratory distress (SRD), and the most critically ill group (CM+SRD). Homogeneity of the frequencies of *KIR* genes among different disease entities was assessed using Chi-square or Fisher's exact test, where indicated. F: Fisher's exact test, n.a: not applicable.

Killer-cell Immunoglobulin-like Receptors (*KIR*) and malaria caused by *Plasmodium falciparum* in The Gambia

Louis-Marie Yindom[1*], Robert Forbes[1], Peter Aka[1], Omar Janha[1], David Jeffries[1], Muminatou Jallow[1], David J. Conway[1], Michael Walther[1]

Medical Research Council Laboratories, Fajara, Banjul, The Gambia[1]
E-mail: louis-marie.yindom@ndm.ox.ac.uk

Journal: Tissue Antigens

A

Centromeric genotype	3DL3	2DS2	2DL2	2DL3	2DL5	2DS3/5	2DP1	2DL1	Motif content and description	UM n (%)	SM n (%)
c-A/A	■	□	□	■	□	□	■	■	Homozygous for c-A motif	20 (10.6)	20 (15.0)
c-B2/B2	■	■	■	□	■	■	■	■	Homozygous for c-B2 motif	24 (12.8)	16 (12.0)
c-B3/B3	■	□	□	■	■	■	■	■	Homozygous for c-B3 motif	2 (1.1)	1 (0.8)
c-A/B2	■	■	■	■	■	■	■	■	Heterozygous c-A and c-B2 motifs	60 (31.9)	41 (30.8)
c-A/B1	■	■	■	■	□	□	■	■	Heterozygous c-A and c-B1 motifs	21 (11.2)	10 (7.5)
c-A/Bx1	■	□	■	■	□	□	■	■	c-A + B motif lacking 2DL5 and all centromeric activating genes	11 (5.9)	9 (6.8)
c-A/Bx2	■	■	■	■	■	□	■	■	c-A + B motif lacking at least two genes including 2DS3 and 2DS5	16 (8.5)	9 (6.8)
c-A/Bx3	■	■	■	■	□	■	■	■	c-A + B motif lacking one or more genes including 2DL5	9 (4.8)	7 (5.3)
c-A/Bx4	■	□	■	■	■	■	■	■	c-A + B motif lacking one or more genes including 2DS2	7 (3.7)	9 (6.8)
c-A/Bx5	■	□	■	■	□	■	■	■	c-A + B motif lacking at least two genes including 2DS2 and 2DL5	7 (3.7)	4 (3.01)
c-A/Bx6	■	□	□	■	□	■	■	■	c-A + B motif similar to B3 but lacking one or more genes including 2DL5	0 (0.0)	2 (1.5)
c-A/Bx7	■	□	■	■	■	□	■	■	c-A + B motif lacking 2DS2, 2DS3 and 2DS5	4 (2.1)	1 (0.8)
c-A/Bx8	■	□	□	■	■	□	■	■	c-A + B motif lacking 2DL2 and all centromeric activating genes	1 (0.5)	0 (0.0)
c-B1/Bx9	■	■	■	□	■	■	□	■	c-B1 + B motif similar to B2 but lacking 2DP1	4 (2.1)	1 (0.8)

B

Telomeric genotype	2DL4	3DL1	3DS1	2DS1	2DS4	3DL2	Motif content and description	UM n (%)	SM n (%)
t-A/A	■	■	□	□	■	■	Homozygous for t-A motif	135 (71.8)	90 (67.7)
t-A/B1	■	■	■	■	■	■	Heterozygous t-A and t-B1 motifs	11 (5.9)	9 (6.8)
t-A/Bx1	■	■	□	■	■	■	t-A + B motif lacking at least one gene including 3DS1	31 (16.5)	25 (18.8)
t-A/Bx2	■	■	■	□	■	■	t-A + B motif lacking at least one gene including 2DS1	11 (5.9)	9 (6.8)

Supplementary Figure 1. The KIR centromeric (A) and telomeric (B) genotypes and their motif content. Filled box means gene is present and open box means the gene is absent. c-A: centromeric A motif, c-B: one of the known centromeric B motifs, c-Bx: one of the new B motifs. UM: uncomplicated malaria, SM: severe malaria, n: number of individuals carrying the genotype of interest. Rare genotypes present in less than 1% of the studied population are not included.

Killer-cell Immunoglobulin-like Receptors (*KIR*) and malaria caused by *Plasmodium falciparum* in The Gambia

Louis-Marie Yindom[1*], Robert Forbes[1], Peter Aka[1], Omar Janha[1], David Jeffries[1], Muminatou Jallow[1], David J. Conway[1], Michael Walther[1]

Medical Research Council Laboratories, Fajara, Banjul, The Gambia[1]
E-mail: louis-marie.yindom@ndm.ox.ac.uk

Journal: Tissue Antigens

a)

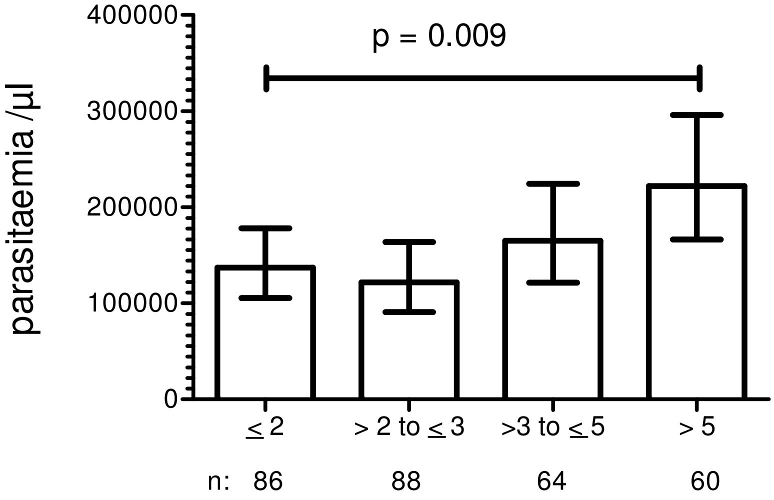

b)

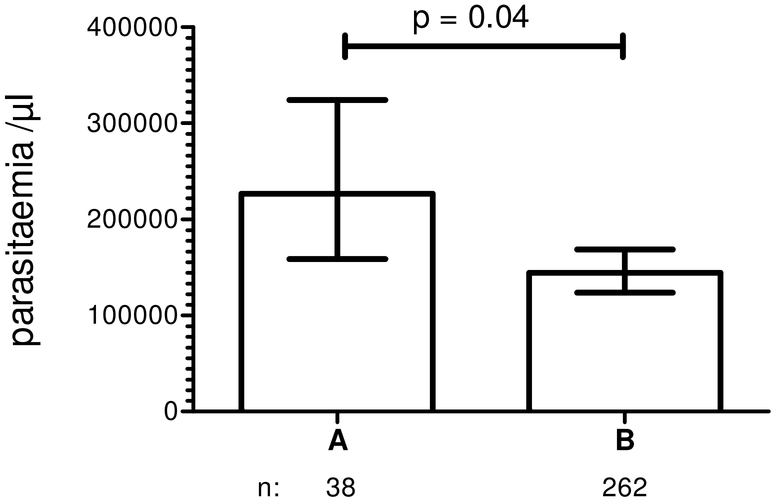

Supplementary Figure 2. a) Ratio of inhibitory / activating KIR genes and b) KIR genotypes associate with level of parasitaemia in the blood. Bars represent the geometric mean with 95% CI, n: number of individuals with blood parasitaemia data, p-value in 2a) refers to test for linear trend and p-value in 2b) refers to t-test, both performed on log transformed data.
